# Maternal Satisfaction with Perinatal Care and Breastfeeding at 6 Months Postpartum

**DOI:** 10.3390/ijerph22081246

**Published:** 2025-08-09

**Authors:** Caitlin M. Dressler, Karina M. Shreffler, Ingrid R. Wilhelm, Jameca R. Price, Karen P. Gold

**Affiliations:** 1 Fran and Earl Ziegler College of Nursing, University of Oklahoma Health Sciences Center, Oklahoma City, OK 73117, USA; caitlin-littlejohn@ouhsc.edu (C.M.D.); ingrid-wilhelm@ouhsc.edu (I.R.W.); 2 Department of Obstetrics and Gynecology, The University of Oklahoma School of Community Medicine, Tulsa, OK 74135, USA; jameca-price@ouhsc.edu (J.R.P.); karen-gold@ouhsc.edu (K.P.G.)

**Keywords:** breastfeeding, perinatal care, patient satisfaction, pregnancy, birth

## Abstract

Positive childbirth experiences increase breastfeeding in the early postpartum period. Using a diverse, clinic-based sample of predominately low-income women (n = 118) recruited at their first prenatal appointment in 2017–2018 and followed through six months postpartum, binary logistic regression analyses were used to examine the association between maternal satisfaction with perinatal care measured two weeks postpartum and breastfeeding at six months postpartum. Participants reported high satisfaction with perinatal care overall (mean = 25.7; range of 6–30), and 25% of participants reported breastfeeding at six months postpartum. Regression results found that greater satisfaction with perinatal care is associated with higher odds of breastfeeding at six months postpartum (OR = 1.19; *p* < 0.05), controlling for sociodemographic characteristics. These findings that have important implications for providers as they identify a group at risk for shorter breastfeeding duration: those who are less satisfied with their perinatal care. More research is needed to identify methods providers can use to increase satisfaction with care as well as to successfully encourage and assist mothers with breastfeeding despite challenges that might arise during pregnancy or childbirth that are associated with low satisfaction.

## 1. Introduction

The American Academy of Pediatrics (AAP) and World Health Organization (WHO) recommend exclusive breastfeeding for the first six months of life and continued breastfeeding through the first two years of life while transitioning to solid foods [1,2]. Despite the recommendation to exclusively breastfeed for six months, only about 25% of new mothers obtain this goal in the United States [3]. Breastfeeding is well-known to improve the short- and long-term health of both women and their children [4,5]. Breastfeeding that meets recommended exclusivity and duration is often a goal of childbearing women and their healthcare providers [6]. Supporting women to initiate and sustain breastfeeding through at least six months is critical for public health [7,8].

Research examining risks for early cessation of breastfeeding often focuses on hospital practices, cultural differences, or personal characteristics that might not be modifiable such as age, ethnicity, socioeconomic status, and maternal employment [9]. To facilitate sustained breastfeeding, it is critical to identify predisposing influences on breastfeeding duration that are susceptible to intervention [10]. The influence of maternity care practices is a continued area of foci and surveillance via the CDC’s national survey of Maternity Practices in Infant Nutrition and Care (mPINC), and the annual CDC Breastfeeding Report Card [3,11]. While there is expansive literature investigating the influence of perinatal care practices and a variety of psychosocial predictors of breastfeeding [12,13,14,15,16], there are only a few that examine how maternal satisfaction with perinatal experiences are related to long-term breastfeeding success [17,18,19,20].

The perinatal period and childbirth experience can have an important impact on the physical and mental health of the mother and the wellbeing of the family [21,22,23]. The care that physicians and nurses provide during the prenatal period, childbirth, and immediately after impact the experience a woman attributes to the birth of her child [22,24,25]. Positive experiences lead to greater maternal self-efficacy, confidence, competence, and decreased levels of stress and anxiety [25,26]. A woman whose perinatal care experiences support psychosocial development that closely aligns with her needs results in neurocognitive changes that result in richer, more supportive environments for the infant [25]. Negative childbirth experiences, on the other hand, can lead to feelings of powerlessness [27], increased levels of stress [28], and depression [29]. These can often alter the interactions between a mother and her child [30]; postnatal depression, for example, can have long-term consequences for infants’ impaired cognitive and emotional development [31].

Positive childbirth experiences [32,33] and hospital practices encouraging breastfeeding within the first hour after birth, exclusive breastmilk feeding while in the hospital, rooming in, no pacifier unless medically indicated, and community postpartum lactation support [3,11,13,15,24,34] increase breastfeeding during the early postpartum period. Less is known, however, regarding the association between maternal satisfaction with care across the perinatal period and sustained breastfeeding outcomes. Yet, it is clear that providers have the potential to directly influence breastfeeding tendencies in mothers [9]. When non-medically indicated supplementation with formula is recommended by the pediatrician, women are more likely to cease exclusive breastfeeding [14,35]. Alternatively, supportive providers increase breastfeeding duration by educating women about current national goals and recommendations for breastfeeding duration [3,36]. Patients often rely on their healthcare professionals to provide recommendations regarding their health behaviors that will benefit their overall health and offer solutions when they experience difficulties. This relationship is often built over time between the patient and provider. The quality of communication between the healthcare professional and a mother can impact the satisfaction of care received during the childbirth experience [37], and it can have a direct influence on breastfeeding self-efficacy and subsequent duration [38]. During pregnancy and childbirth, the patient relies on her provider to provide the most recent evidence-based care. During this critical time, providers are positioned to support mothers and facilitate the development of optimum breastfeeding behaviors through interactions that may influence new mothers’ satisfaction with care.

The present study uses longitudinal data to examine the causal impact of maternal satisfaction with perinatal care following childbirth and breastfeeding at six months postpartum. We expect that mothers with greater satisfaction with care will have higher odds of breastfeeding at six months postpartum. The results of the study are vital to inform healthcare practices that have the potential to improve maternal satisfaction with perinatal care and increase the odds of continued breastfeeding into the postpartum period.

## 2. Materials and Methods

This study applied quantitative analysis of longitudinal survey data to explore the association between maternal satisfaction with perinatal care and breastfeeding at six months postpartum. This study draws from a diverse and primarily low-income sample of 177 mothers, aged 16–38, recruited from two university-affiliated women’s health clinics in a metropolitan area in the South-Central United States. The goal of the larger study was to identify risk and protective factors for maternal and child health outcomes. Participants were recruited at their first prenatal visit in 2017–2018 and followed through their pregnancies and after delivery to 22 months postpartum, participating in up to nine survey assessments. The sample for the current study includes the 118 participants who continued participation through the sixth assessment, occurring at six months postpartum. A majority of the participants reported a historically marginalized racial/ethnic identity (40% non-Hispanic White (n = 47); 28% Black (n = 35); 13% Hispanic (n = 16); 17% Native American (n = 20)), and nearly 90% reported using public insurance to pay for their perinatal care.

Following approval from the Institutional Review Boards from the two participating universities, potential participants were approached based on screening questions by nursing staff members. Screening items included being 16 weeks pregnant or less, planning to continue the pregnancy and be a primary caregiver, and being between 15 and 45 years of age. Those who were potentially interested in participating were transferred to members of the research team, who informed potential participants of the study’s purpose and procedures and signed informed consent (or assent and parental consent from participants under 18) obtained from those who wished to participate. Following enrollment, participants were texted survey links via REDCap and compensated between $25–50 USD each time surveys were completed.

Measures for this study come from the first, fourth, and sixth assessment, conducted at the first prenatal visit, approximately two weeks post-birth, and six months post birth, respectively. The primary independent variable, satisfaction with perinatal care, was assessed at two-weeks post-birth via a six-item survey that is a section of the Canadian Maternity Experiences Survey (MES) [39]. The development of the MES was initiated by the Canadian Perinatal Surveillance System with the goal of surveying new mothers on the experiences surrounding pregnancy, birth and immediate postpartum period. The MES questionnaire has been tested for reliability and validity and provides high quality data regarding women’s childbirth experiences [39]. The section of the survey used for this study asked participants to rank their overall satisfaction with the interactions with their healthcare providers regarding the information given to them, compassion and understanding, competency, the concern for their privacy, the respect shown to the patient, and the patients’ involvement in making decisions with the healthcare provider [40]. Six items are used to assess satisfaction with perinatal care on a 5-point Likert scale, with scores that range from 6 to 30, with 6 indicating low satisfaction and a score of 30 signaling high satisfaction. This sample also ranged from 6 to 30, with a Cronbach’s alpha of 0.96, indicating high reliability of the scale.

Sustained breastfeeding, the dependent variable of this study, was measured at 6 months postpartum as a dichotomous variable (1 = yes; 0 = no) based on the survey question, “Are you currently breastfeeding?”

Several sociodemographic variables previously associated with breastfeeding behaviors were used as control variables for this study and were collected at the participant’s first prenatal appointment. These include maternal age measured in years. Marital status was coded as a dichotomous variable where 1 = married and 0 = single. Race/ethnicity was coded into dummy variables using Census priority coding guidelines for Native American, Hispanic, and Black participants, with non-Hispanic White as the reference category; and parity coded from 0 to 9 children. Due to little variation in participants’ income levels with nearly 90% reporting public insurance, income was not included as a control variable for the study. Instead, we used an indicator of economic hardship; participants were asked if they experienced hardships in the past 12 months, such as homelessness, food insecurity, and not being able to pay for utility bills. “Yes” responses were coded as a 1 and summed to create an index of economic hardship ranging from 0 to 7.

Binary logistic regression analyses were used to examine the association between maternal satisfaction with perinatal care measured two weeks postpartum and breastfeeding at six months postpartum, controlling for maternal age, economic hardship, race/ethnicity, marital status, and number of children.

## 3. Results

Descriptive statistics are included in Table 1. Approximately 25% of participants reported continuing to breastfeed at six months postpartum. Although the range of satisfaction with perinatal care spanned the full range, the average score was quite high (M = 25.73; SD = 4.91), indicating fairly high satisfaction with perinatal care on average. As mentioned above, the sample was comprised of a majority of participants from historically disadvantaged racial/ethnic and income status groups. On average, participants experienced nearly two economic hardship events in the past year (M = 1.91; SD = 2.23), and 28% reported being married at the time of the first survey assessment. On average, participants had at least one child (M = 1.3; SD = 1.5).

**Figure 1:**
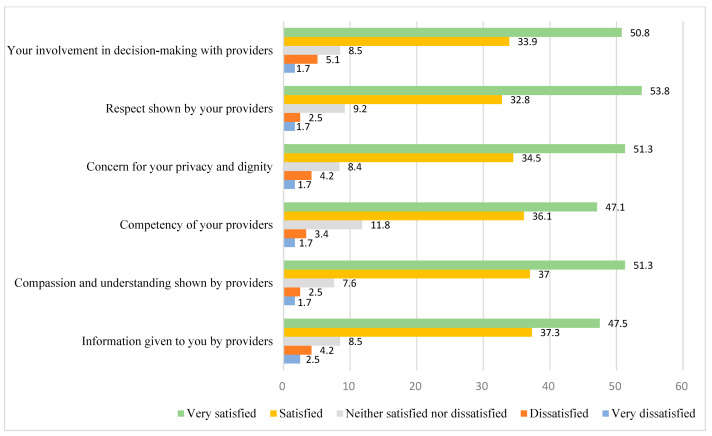
Distribution of Responses to Satisfaction with Perinatal Care Providers Items.

Approximately half of participants rated their satisfaction with their perinatal care providers as “very satisfied” on each of the scale items. See Figure 1 for the distribution of responses to the satisfaction with perinatal care scale items.

The logistic regression analysis (see Table 2) examined the association between satisfaction with perinatal care following birth and the odds of sustained breastfeeding at six months postpartum. We found that greater satisfaction with perinatal care was associated with higher odds of continued breastfeeding at six months postpartum (OR = 1.19; *p* < 0.05), controlling for maternal age, economic hardship, race/ethnicity, marital status, and number of children. Marital status (OR = 2.83; *p* < 0.05) also was associated with increased odds of breastfeeding at six months postpartum, but we did not find other significant associations between control variables and odds of breastfeeding.

## 4. Discussion

This study supports our hypothesis that maternal satisfaction with her prenatal and childbirth care is related to her likelihood of continued breastfeeding at six months postpartum. Although a considerable body of research has investigated predictors of breastfeeding previously, most studies focused on breastfeeding initiation or relied on retrospective reports of breastfeeding duration, resulting in overestimation of duration [41]. Additionally, prior studies have examined how baby friendly hospital practices influence breastfeeding outcomes, but they did not emphasize maternal perceptions of satisfaction with her perinatal care. The present study shows the importance of maternal perceptions of healthcare providers and patient interactions for successful breastfeeding behaviors in a diverse and predominately low-income sample. The relatively low rate of continued breastfeeding at six months—25% of participants in our sample—is comparable to rates among similar samples, such as mothers living in the South, those who report Black racial identity, and those living below the poverty line [42].

This study highlights that perinatal care and interactions between healthcare providers and their patients are critical to facilitating optimum breastfeeding behaviors. This is a similar finding to others that have found that perinatal care is critical for breastfeeding initiation and exclusivity in the early postpartum period. For example, breastfeeding withing the first hour of life has been associated with greater self-efficacy and duration of breastfeeding [38]. Early initiation of breastfeeding is still a barrier for healthcare staff following the delivery of a newborn. The World Health Organization/UNICEF has administered the Baby-Friendly Hospital Initiative (BFHI) with the development of the 10 Steps to Successful Breastfeeding [34]. This initiative was developed to assist healthcare facilities that care for women and children to implement healthcare practices that protect and promote breastfeeding. Studies on the effectiveness of BFHI found that hospital staff showed competence in explaining the benefits of and how to manage breastfeeding [37,43]; and mothers roomed in with the infant, the infant was only given breastmilk, and pacifiers were not used [15]. Despite the BFHI program being initiated over 25 years ago, these practices have yet to be standardized as a public health policy and only 26.86% of annual U.S. births occur in BFHI-designated hospitals [44]. Maternal perceptions of support for breastfeeding are higher when hospitals are designated as Baby-Friendly [45], but prior studies have not yet examined the association between BFHI-designation and broader maternal satisfaction with perinatal care. Future research is needed to examine whether the mechanism underscoring the influence of BFHI on breastfeeding outcomes might be due in part to maternal perceptions of care and the provider–patient relationship.

### 4.1. Strengths and Limitations

This study fills a gap in the literature by exploring the importance of maternal satisfaction with perinatal care on continued breastfeeding at six months postpartum. The current study has several notable strengths, including a diverse sample of low-income women recruited during pregnancy and followed into the postpartum period. The sample predominately comprises participants with demographic characteristics associated with considerably lower rates of breastfeeding than the national average [42]. Identifying factors associated with breastfeeding at six months postpartum among this sample is important for future intervention efforts among similar populations. Further, rather than relying on retrospective reports of satisfaction with care or breastfeeding duration, the study is prospective and captures maternal satisfaction with perinatal care following birth and current breastfeeding at six months postpartum.

Despite its strengths, this study has several limitations due to study assessments that should be addressed in future research. First, the measure of satisfaction with perinatal care was only assessed following birth and did not specify specific care providers. Future research should examine maternal perceptions of perinatal care across the prenatal period, not only following childbirth. Additionally, the measures should ask if responses are given in reference to one key provider or multiple providers. Second, our data only included a measure of continued breastfeeding at six months postpartum as opposed to exclusivity of breastfeeding or a calendar. Future research should examine exclusivity as well as duration of breastfeeding beyond six months postpartum. It might be useful to employ event history analysis to examine duration of breastfeeding, which would enable a more sensitive analysis of breastfeeding duration.

### 4.2. Implications of Study Findings

This study has important implications for healthcare system practices and for providers who work with women during pregnancy. Although there has been considerable attention on the importance of hospital practices for breastfeeding outcomes as noted above, our results highlight the need for greater emphasis on provider–patient interactions and communication. Maternal satisfaction with various domains of perinatal care including information, compassion, and respect shown by providers has long-term implications for breastfeeding outcomes. Ensuring that providers are aware of the long-term benefits of a positive provider–patient relationship can help promote outcomes such as breastfeeding that endure beyond the childbirth experience.

## 5. Conclusions

Positive childbirth experiences increase breastfeeding behaviors in the first six months of the postpartum period. Breastfeeding has numerous lifelong health benefits for both mothers and newborns and should be promoted by perinatal healthcare providers. This study found that maternal satisfaction with prenatal and childbirth care predicted greater odds of sustained breastfeeding through six months postpartum. This study highlights the importance of provider interaction, communication, and caregiving behaviors for breastfeeding outcomes. As breastfeeding duration continues to fall below national recommendations, identifying and promoting caregiving practices that promote sustained breastfeeding are an important target for intervention.

## Figures and Tables

**Table 1:** Descriptive Statistics of Study Variables (N=118).

Variables	M or %	SD	Range
Breastfeeding (6 mo)	25%		0–1
Satisfaction with care scale	25.73	4.91	6–30
Satisfaction with:			
Information provided by providers	4.24	0.94	1–5
Compassion shown by providers	4.33	0.85	1–5
Competency of providers	4.25	0.89	1–5
Concern for privacy/dignity	4.28	0.94	1–5
Respect shown by providers	4.36	0.86	1–5
Your involvement in decision-making	4.27	0.94	1–5
Sociodemographic characteristics			
Age	25.67	0.37	16–38
Economic hardship	1.91	2.23	0–6
Married	28%		0–1
Race/ethnicity			
White	40%		0–1
Black	28%		0–1
Hispanic	14%		0–1
Native American	17%		0–1
Parity	1.30	1.50	0–10

**Table 2:** Odds of Breastfeeding at 6 Months Postpartum by Satisfaction with Perinatal Care and Sociodemographic Characteristics (N = 118).

Variables	Odds Ratio (95% CI)		SE
Satisfaction with Perinatal Care	1.19 (1.04—1.37)	*	0.07
Sociodemographic characteristics			
Age	1.02 (0.92—1.13)		0.05
Economic hardship	0.88 (0.66—1.17)		0.15
Married	2.83 (0.99—8.09)	*	0.54
Race/ethnicity (White)			
Black	0.40 (0.11—1.49)		0.67
Hispanic	1.26 (0.30—5.34)		0.74
Native American	0.63 (0.14—2.80)		0.76
Parity	1.21 (0.87—1.67)		0.17
Constant	0.01	**	2.38
Nagelkerke R Square	0.28		

Notes: * *p* < 0.05; ** *p* < 0.01. Reference category in parentheses.

## Data Availability

The data presented in this study are available from the authors on request.
